# Diagnosis of *Fusarium* Infections: Approaches to Identification by the Clinical Mycology Laboratory

**DOI:** 10.1007/s12281-015-0225-2

**Published:** 2015-07-01

**Authors:** Anne D. van Diepeningen, Balázs Brankovics, Jearidienne Iltes, Theo A. J. van der Lee, Cees Waalwijk

**Affiliations:** CBS-KNAW Fungal Biodiversity Centre, Uppsalalaan 8, 3584 CT Utrecht, The Netherlands; Institute of Biodiversity and Ecosystem Dynamics, University of Amsterdam, Amsterdam, The Netherlands; Plant Research International Wageningen UR, Droevendaalsesteeg 1, 6708 PB Wageningen, The Netherlands

**Keywords:** *Fusarium*, Multi-locus sequence typing, Clinical observations, DNA-based diagnostic tools, Peptide-based diagnostic tools, Antifungal susceptibility

## Abstract

Infections caused by the genus *Fusarium* have emerged over the past decades and range from onychomycosis and keratitis in healthy individuals to deep and disseminated infections with high mortality rates in immune-compromised patients. As antifungal susceptibility can differ between the different *Fusarium* species, identification at species level is recommended. Several clinical observations as hyaline hyphae in tissue, necrotic lesions in the skin and positive blood tests with fungal growth or presence of fungal cell wall components may be the first hints for fusariosis. Many laboratories rely on morphological identification, but especially multi-locus sequencing proves better to discriminate among members of the species complexes involved in human infection. DNA-based diagnostic tools have best discriminatory power when based on translation elongation factor 1-α or the RNA polymerase II second largest subunit. However, assays based on the detection of other fusarial cell compounds such as peptides and cell wall components may also be used for identification. The purpose of this review is to provide an overview and a comparison of the different tools currently available for the diagnosis of fusariosis.

## Introduction

Taking the genus *Fusarium* in its currently broadest definition [1•], it contains many species with either saprophytic or plant pathogenic lifestyles. However, the genus also contains species capable of forming a plethora of mycotoxins and, more importantly, harbours species capable of causing opportunistic infections in human and animals [2, 3•]. In recent years, the numbers of infections reported to be caused by *Fusarium* species have increased [4, 5].

Many species within the genus *Fusarium* that were recognized based on morphological characters proved to be species complexes, with little to no morphological differences, rather than single species. For their recognition, often multi-locus sequence typing (MLST) is required [6–9]. Most of the identified opportunistic *Fusarium* pathogens belong to the *Fusarium solani* species complex (FSSC), the *Fusarium oxysporum* species complex (FOSC) and *Fusarium fujikuroi* species complex (FFSC). Less frequently encountered are members of the *Fusarium incarnatum-equiseti* (FIESC), *Fusarium dimerum* (FDSC) and *Fusarium chlamydosporum* species complexes (FCSC) or species such as *Fusarium sporotrichioides* [8, 10, 11]. Some of these species cause infections worldwide, but other species appear to be endemic in certain areas [12, 13].

*Fusarium* species cause a broad spectrum of opportunistic infections in humans. In otherwise healthy individuals, the most prevalent fusarioses are onychomycosis and skin infections—that due to their lack of urgency often go undiagnosed—and keratitis that especially in warmer, drier climates can reach high incidences. In immunocompromised patients, deep invasion at the primary site of infection can occur, while especially in patients with haematological disorders, disseminated infections occur [3•, 14]. In the case of disseminated fusariosis, the manifestation of multiple necrotic lesions spread over the body and positive blood cultures are highly suggestive of fusariosis.

*Fusarium* strains have high levels of intrinsic antifungal resistance: Recently, diagnostic guidelines recommend amphotericin B (AMB) and voriconazole (VOR) as the preferred drugs of choice for treatment of deep and disseminated infections and recommend against the use of echinocandins [15•]. Although AMB and VOR are sufficient for the treatment of the majority of *Fusarium* infections, some *Fusarium* species are not susceptible to AMB and VOR or posaconazole (POS) [16, 17]. In many countries, fungal keratitis is treated with natamycin, that is effective against most but not all, *Fusarium* species, e.g. [18]. Detailed studies on antifungal susceptibility profiles emphasize the need for species-level identification for best-adapted treatment strategies [5].

This review gives an overview of the currently available techniques for the diagnosis of fusarioses and for the identification of *Fusarium* as opposed to other fungal species and within the species to species complex or preferably species level. Here, we also discuss whether each diagnostic technique is suitable for direct detection of *Fusarium* in patient specimens or whether it is restricted to identification of a cultured isolate.

### Clinical Observations Indicative for Fusariosis

Some clinical observations may give a first hint to *Fusarium* being the etiological agent causing an infection:

#### Direct Microscopy in Keratitis

In vivo confocal microscopy of the infected eye is a non-invasive and rapid technique to determine whether there is a fungal or other microbial infection and has become part of the routine especially in countries where there is a high incidence of fungal keratitis [19–21]. Sensitivity and specificity of fungal and acanthamoebal diagnosis by confocal microscopy are estimated at 88.3 and 91.1 %, respectively [21]. Even the modern smartphone camera and pocket magnifier can now be used to detect fungal hyphae in corneal scrapings [22]. However, fungal structures that enable genus or even species-level identification are seldom formed in clinical specimens [20].

#### Blood Tests in Disseminated Infections

Members of the genus *Fusarium* are among the few filamentous fungal species yielding positive blood cultures, while it is known for decades that such disseminated fusariosis is often accompanied by multiple necrotic skin lesions [23, 24]. Less well known is the fact that the serum galactomannan tests developed for *Aspergillus* infections can also cross-react with *Fusarium* infections, since both genera have a similar cell wall structure and hence may be an indication for disseminated fusariosis [16, 25]. The Platelia *Candida* antigen detection enzyme immunoassay directed against mannan has been reported to give cross-reactions with *Fusarium verticillioides*, but not with *F. solani* or *F. oxysporum* [26]. Also the presence of beta-glucan is not specific for particular fungal genera, and *Fusarium* spp. do produce this cell wall component [27].

#### Radiology

Radiological findings may be helpful for the identification of infective fungal diseases, but they are often not specific for a given species/etiological agent, as many times the findings include alveolar and interstitial infiltrates, nodules and cavities that are typical for a wide range of diseases. Studying pulmonary fusariosis, Marom et al. [28] showed that nodules and lung masses are seen in 82 % of the patients on CT scans but only in 45 % on chest radiography. Non-specific findings were observed in 30 % of the patients, while findings at presentation were observed in 25 %. Halo and reverse halo signs are absent in *Fusarium* infections [28, 29]. Early chest CT imaging in immunocompromised patients suspected of having invasive fungal pneumonia can help identify disease early, leading to improved outcome [30].

### Histology/immunohistochemistry

Confirmatory diagnosis of fusariosis by histopathology is strongly recommended by the European Confederation of Medical Mycology and European Society of Clinical Microbiology and Infectious Diseases (ECMM/ESCMID) guidelines [15•]. In tissue, the hyphae of *Fusarium* are similar to those for instance *Aspergillus* species, with hyaline and septate filaments that typically dichotomize in acute and right angles [14]. Adventitious yeast-like sporulation may be present in tissue, and the finding of hyphae and yeast-like structures together is highly suggestive of fusariosis in the high-risk population [14]. Some *Fusarium* species may form chlamydospores in the hyphae in tissue [31]. However, overall, directly observed morphological characteristics in histopathological examination are non-specific.

Alternate techniques have been used to determine the specific agent: Immunohistochemistry uses antibodies directed at fungal cell antigens—mainly proteins—to identify organisms visualized in tissue. Advantages of immunohistochemistry include relatively rapid results, preservation of tissue morphology and the ability to use formalin-fixed paraffin-embedded (FFPE) tissue. However, few antibodies are commercially available for specific moulds [32], but some authors have described the more or less specific detection of *Fusarium* in human tissue with in-house-made polyclonal fluorescent or peroxidase-linked antibodies, e.g. [33, 34].

In the case of in situ hybridization (ISH), the target for recognition is DNA or RNA, while as probe, a labelled complementary strand is used. Several types of probes have been successfully used for the detection of *Fusarium* strains: oligonucleotide DNA [35, 36], PNA (peptide nucleic acids; DNA mimics with a peptide backbone) [37, 38] and LNA (a mix of DNA and locked nucleic acid (LNA)-modified nucleotides in which the 2′ oxygen and the 4′ carbon are linked through a methylene unit) [39]. Especially, the PNA and LNA nucleotides hybridize strongly to their complementary RNA and DNA nucleotides, producing hybrids that are thermally stable. Probes can be radio-, fluorescent- or antigen-labelled. Most probes developed for *Fusarium* species have been directed at the ribosomal RNA and have low levels of specificity.

Once fungal elements have been detected in patient tissue, there is also the option to use PCR-based tools (see below) for their identification. A restriction here is that sufficient pure fungal DNA needs to be isolated from the sample. Several commercial kits are available to isolate DNA from FFPE tissues where deparaffinization can be obtained via xylene or microwave irradiation [40]. Lau et al. [41] provide an example of successful pan-fungal PCR and sequencing both in fresh and FFPE tissue specimens from patients with IFI. Laser capture microdissection (LCM) can allow for the microscopic procurement of specific cell types from FFPE tissue sections for specific further analyses [42].

### Morphology-based Identification

Identification of fungal isolates has long been based on morphological characteristics of cultures, and for many laboratories, this is still the standard for identification. However, many *Fusarium* species look similar in culture and have been shown to represent species complexes instead of single species. Often colonies obtained from patients prove degenerate in their colony morphology or pionnotal [2, 43]. The media and growth conditions used influence growth rate and colony pigmentation, formation of differentiating structures and the dimensions of these structures. The formation of specific structures may take weeks and may require UV or daylight induction to be formed at all.

The Atlas of Clinical Fungi [43] provides a dichotomous key for the morphological identification of clinical fungi till date, including *Fusarium*. Guarro and Gené in their 1992 paper [44] detailed about the differences between *Fusarium*, *Acremonium* and *Cylindrocarpon*, but Summerbell and Schroers [45] proved some *Acremonium* and *Cylindrocarpon* species are actually also members of the genus *Fusarium*. Azor et al. [46] provide a key with morphological features for clinical *Fusarium* species that agrees well with molecular identification based on sequences of the beta-tubulin gene. For the identification of *Fusarium* species in general—not just known clinical strains—Leslie and Summerell’s text [2] provides a convenient tool. Each key uses different preferred media.

### DNA-based Identification Techniques

#### PCR-based

PCR assays are the most widely used method for the amplification of DNA in laboratories. The exponential amplification of target DNA makes it possible to detect even small amounts of fungus, while target choice makes a test more or less specific. Many PCR-based tools have been used for the identification of fungal infections, but only few tools are commercially available, and they depend on in-house validation. Recent guidelines [15•] recommend the use PCR-based diagnostic tools when available for confirmation of infection.

A PCR detection based on the intergenic spacer (IGS) region has been developed for different agricultural important *Fusarium* species (complexes) that can also distinguish clinical species complexes like *Fusarium equiseti* and *F. sporotrichioides* because different-sized fragments are produced [47]. A fluorescent PCR fragment length analysis based on the internally transcribed spacer 2 (ITS2) region is available to distinguish between *Aspergillus*, *Candida* and *F. oxysporum* [48], but the method cannot distinguish between the *Fusarium* spp. that have (near) identical ITS amplicon lengths. Utilization of a semi-nested PCR (nPCR) increases the sensitivity of this method and makes it usable in blood samples with a low pathogen load [48]. Both Ahmad et al. [49] and Sugawara et al. [50] used nPCR—based on the ribosomal ITS1-5.8S-ITS2 region—to detect *Fusarium* in bronchoalveolar lavage (BAL), serum and/or blood samples. However, for both authors’ research, the chosen DNA region limits identification to genus level.

In rep-PCR, non-coding repetitive extragenic palindromic (REP) sequences interspersed throughout the fungal genome are amplified using PCR. Initial discovery of REP elements occurred in the genomes of *Escherichia coli* and *Salmonella*. REP elements are generally between 33 and 40 bp in length, and the amplified DNA fragments, when separated by electrophoresis, constitute a genomic fingerprint that can be employed for subspecies discrimination and strain delineation of bacteria and fungi. DiversiLab is a commercial automated repetitive sequence-based PCR method that has been tested for a set of 26 *Fusarium* isolates, only two thirds of which could be identified similarly to sequence-based identification on the large ribosomal subunit (LSU) and elongation factor 1-α [51].

In multiplex PCR, several DNA targets are amplified simultaneously. To distinguish between different genera of human pathogens on one side and opportunists like *Candida*, *Cryptococcus* and *Fusarium* on the other, a multiplex tandem PCR on cultures was developed based on ITS, beta-tubulin and elongation factor 1-α regions [52]. A multiplex PCR based on one gene, galactose oxidase B (*gaoB*), is available for the differentiation to the species level of several *Fusarium* maize pathogens that are in fact also human opportunists including *F. verticillioides* and *Fusarium subglutinans* [53].

During real-time or quantitative PCR (qPCR), the target DNA is amplified and simultaneously detected, shortening time to diagnosis. The two most commonly used methods act via non-specific dyes intercalating in the double-stranded DNA or via fluorescently labelled probes. The number of cycles required to detect a signal is an indication of the initial amount of target sequence in the sample. Bernal-Martínez et al. [54] used the ribosomal DNA region for development of a duplex qPCR capable of detecting *F. solani* and non-*F. solani* species. Testing in a murine model showed high sensitivity and low detection limits, though better for *F. solani* than for non-solani species tested. Muraosa et al. [55] used a cycling probe technology-based real-time PCR with a chimaera probe, composed of RNA and DNA targeting the 28S ribosomal DNA (rDNA), for the genus *Fusarium* as well as for FSSC that had a high sensitivity and also worked in serum samples, but not in whole blood samples.

Quantitative multiplex PCRs with labelled primers based on 5.8S and 28S rDNA genes have been described to distinguish between genera of several pulmonary fungal pathogens including *Fusarium*, *Aspergillus* and Mucorales. The essay worked both in isolates and different patient samples [56]. A second multiplex real-time PCR method uses hybridization probes for the detection and the quantification of plant and human pathogenic *Fusarium proliferatum*, *F. subglutinans*, *Fusarium temperatum* and *F. verticillioides* [57].

Furthermore, there are PCR-based tools that are not described for clinical diagnostics such as, e.g. multiplex ligation-dependent probe amplification (MLPA). For studies on the epidemiology, biodiversity and genotyping, amplified fragment length polymorphism (AFLP) analyses [58–61], enterobacterial repetitive intergenic consensus PCR (ERIC-PCR) [61, 62], PCR restriction fragment length polymorphism (PCR-RFLP) [62] and random amplification of polymorphic DNA [60] have been used. However, for diagnostics, these methods would only be applicable with a large enough and validated database.

#### Hybridization-based Techniques

In reverse line blot (RLB) hybridization, a DNA sample is first subjected to (multiplex) PCR and then screened against a set of specific probes linked to a membrane. Wang and colleagues [63] developed a set of primers based on the IGS region of clinically important *Fusarium* strains. Their probe set contained one genus-specific, 13 species complex-specific and 52 species-specific probes, with high specificity and very few cross-reactions. The method therefore allows screening for the presence of multiple species in a single reaction. Results on DNA from cultures were concordant with multi-locus sequence analysis.

In a DNA microarray, probes are attached to a solid surface on microscopic slides. Based on the 18S-ITS1-5.8S region, a DNA microarray following a multiplex PCR was developed distinguishing between 14 fungal pathogens including *F. solani* and *F. oxysporum* in blood, BAL and tissue samples [64]. Based on ITS1 and ITS2, a similar array was made for 24 species including the *F. solani* species complex [65].

Microsphere or liquid phase array analysis combines a multiplex PCR with hybridization to probes linked to fluorescent microspheres, followed by analysis with a flow cytometer (Luminex technology). The newest version of this technique can detect up to 500–1000 different microsphere-linked probes simultaneously. Several assays have been based on the ITS2 region and cover commonly occurring species of genera like *Aspergillus*, *Fusarium* and *Zygomycetes* [66, 67]. Buelow et al. [68] devised an array for the identification of common respiratory fungal pathogens, including *Aspergillus fumigatus*, *Rhizopus microsporus*, *Scedosporium apiospermum* and *F. solani* based on elongation factor 1-α and other genes. O’Donnell et al. [69] based their microsphere array on the *rpb2* gene and used it for discrimination between the *Fusarium* involved in lens-associated keratitis. Detection limits typically range from 0.1 to 1 ng of DNA, depending on the fungus being tested. Luminex Molecular Diagnostics (Toronto, Canada) has analyte-specific reagents (ASRs) available for different clinically important species including one for *Fusarium* that has been tested on patient samples [70].

#### DNA Sequencing

The internal transcribed spacer 1 (ITS1) region has been chosen as a general barcode for fungi [71]. However, this locus does not contain enough variation to distinguish, based on single-gene sequences, between many *Fusarium* species [9]. Pan-fungal primers based on the ITS1 region have been used to amplify and sequence PCR products to identify to the genus level several invasive species including *Fusarium* spp. This technique has also been applied in tissue samples [41]. Also, the D1 plus D2 regions of the nuclear large subunit rDNA are not variable enough to distinguish to species level [6, 72]. The β-tubulin gene (β-*tub*), e.g. [46], and RNA polymerase II second largest subunit *rpb2* [69] and elongation factor 1-α (*ef-1*α) [73] have more discriminatory power.

Multi-locus sequence typing (MLST) is currently seen as the best method for the identification of *Fusarium* isolates to species level [15•]. Different MLST schemes have been proposed for the different species complexes (Table 1). Especially regions of *ef-1*α and RNA polymerase II second largest subunit (*rpb2*) have good discriminatory power and are often used in MLST schemes.

**Table 1:** Applied multi-locus sequence typing (MLST) schemes for the genus *Fusarium* and specific species complexes within the genus

Species complex (SC)	MLST scheme	Reference
*F. chlamydosporum* (FCSC)	ITS, LSU, *ef-1*α, *rpb2*, *cal*	[8]
*F. dimerum* (FDSC)	ITS, LSU, β-*tub*, *ef-1*α	[74]
*F. fujikuroi* (FFSC)	ITS, LSU, β-*tub*, *ef-1*α, *rpb2*, *cal*, mtSSU	[75]
	*ef-1*α, *rpb2*	[17]
*F. incarnatum-equiseti* (FIESC)	ITS, LSU, *ef-1*α, *rpb2*, *cal*	[8]
*F. oxysporum* (FOSC)	IGS, *ef-1*α, mtSSU	[59]
	Five variable mitochondrial intergenic regions	[76]
	IGS, *ef-1*α	[7]
*F. solani* (FSSC)	ITS, LSU, *ef-1*α, *rpb2*	[6]
	ITS, *ef-1*α, *rpb2*	[77]
*F. avenaceum* (FASC)	ITS, IGS, β-*tub* mtSSU	[78]
Genus *Fusarium*	*ef-1*α, *rpb1*, *rpb2*	[9]
Multiple SCs	ITS, LSU, IGS, *ef-1*α, *rpb2*, *cal*, mtSSU	[63]

*Loci*: *ITS* internal transcribed spacer, *LSU* large ribosomal subunit (28S), *IGS* intergenic spacer, *ef-1*α elongation factor 1-α, *β-tub* β-tubulin, *cal* calmodulin, *rpb1* RNA polymerase largest subunit, *rpb2* RNA polymerase II second largest subunit

For the identification based on DNA sequences, we rely on blast searches in reference databases and assume that they are sufficiently complete and contain correctly identified and annotated entries. However, 10–20 % of the entries in public repositories like GenBank are estimated to be incorrectly identified to the species level [79]. To limit incorrect entries, two curated databases have been developed for the genus *Fusarium*: the Fusarium-ID database (http://isolate.fusariumdb.org/ [80]) and the *Fusarium* MLST database (http://www.cbs.knaw.nl/fusarium/ [9]).

#### Isothermal Amplifications

Isothermal DNA amplification techniques have been developed that amplify DNA at a single temperature as opposed to PCR, which requires three different temperatures. Isothermal assays are considered to be simple, cost effective and rapid methods for the detection of specific genomic DNA fragments. Loop-mediated isothermal amplification (LAMP) assays have so far been applied to several *Fusarium* plant pathogens [81, 82]. For LAMP, the detection is based on a set of six primers hybridizing to the target gene and amplification, after which the product can be visualized by a nucleic acid dye, often directly in the reaction tube. Rolling circle amplification (RCA) relies on the formation of a circular DNA molecule when the padlock probe—long oligonucleotides, whose ends are complementary to adjacent target sequences—fits exactly on the target sequence that subsequently can be endlessly transcribed and detected directly in the reaction tube or after gel electrophoresis. Specific RCA probes for the *F. oxysporum* and *F. incarnatum-equiseti* species complexes have been developed [83].

### Peptide-based Identification Techniques

Besides the galactomannan tests described above, much of the enzyme-linked immunosorbent assay (ELISA) work in *Fusarium* has been targeting the specific mycotoxins they are known to produce in planta, but we do not know whether these compounds are also produced in patient tissue or whether these molecules, if detected, derive from food intake. Most antibodies targeting *Fusarium* species themselves are not species-specific and hence could be used to determine a human fusariosis, but no diagnosis to species level can be obtained, e.g. [84–86]. Detection limits for ELISA range from 0.1 to 1 μg of mycelium per millilitre of product tested. Several antibodies and ELISA kits for *Fusarium* are commercially available, but as far as we know, none are used in hospital laboratories.

Matrix-assisted laser desorption/ionization time-of-flight mass spectrometry (MALDI-TOF MS) is an emerging tool for fast identification and classification of cultured microorganisms based on their protein spectra. Different commercial companies supply MALDI-TOF MS systems. Systems like VITEK (bioMérieux, Marcy l'Etoile, France) or Biotyper (Bruker Daltronics Inc, Billerica, MA) come with their own processing software and spectral database with reference strains.

For bacteria and yeasts, the necessary databases are further advanced than those for filamentous fungi. However, the studies done on *Fusarium* species are very promising with success rates of identification to the species level of 82–99 % [87–90]. Nevertheless, growth conditions like type and phase (solid/liquid) of media, age of the examined culture, type of sample with or without spores and the treatment of the fungal sample can influence the resulting spectra [91–94]. The limiting part for the level of identification for a given platform seems to be the reliability and level of identification of the strains used for the database production.

The abovementioned studies on *Fusarium* detection by MALDI-TOF MS analysis were all based on pure cultures. Pan et al. [95] in a study on ocular mycoses reported that with MALDI-TOF MS, the fusarioses could be distinguished from the other etiological agents, while Ananthi et al. [96] showed that human tear protein profiles are influenced by the *Fusarium* pathogen. Studies on *Candida* infections in the blood stream suggest that also in blood samples, direct fungal detection may be possible, e.g. [97].

## Conclusions

Several clinical features can hint to a fusariosis: For disseminated infections, these are positive galactomannan tests and typical necrotic skin lesions. For keratitis, there is the presence of hyaline hyphae, while for superficial skin infections and onychomycosis, no *Fusarium*-specific traits are recognized. Especially for deep and disseminated infections, rapid diagnosis is of the utmost importance for timely and adequate treatment. Hence, we see a shift in diagnostic tools applied in the hospital laboratory from classic, morphological determination of the etiological agent that sometimes involves prolonged culturing to obtain all necessary structures for species identification to faster DNA or peptide-based diagnostic tools.

After culturing of *Fusarium* from a patient, MLST identification is currently seen as the best option for species-level identification [15•]. However, if these specific tools are not available in the laboratory, the use of another molecular and/or peptide-based tool is recommended [15•]. Techniques like microsphere/liquid phase arrays and MALDI-TOF may prove faster in making a species-level diagnosis. Combining available tools will enhance the specificity of identification [98].

*Fusarium* also contain many other compounds beside DNA, peptides and cell wall components that could be used for future diagnostic purposes. Volatile organic compound profiles detected by selected ion flow tube-mass spectrometry (SIFT-MS) may have diagnostic value [99]. Detection of toxic or non-toxic secondary metabolites produced in cultures may be another option [100, 101]. No doubt new techniques will evolve that will make diagnosis even faster and more reliable.

The main goal of diagnostic tools is better survival and recovery chances for the patient by selecting adequate treatment. As survival rates in invasive infections greatly depend on early diagnosis, tools directly applicable in patient samples will become more and more important. Recent research on differences in antifungal susceptibility between species and isolates, e.g. [5, 13], demonstrates the need of species-level identification in the case of a fusariosis.
